# Feedlot performance, rumen and cecum morphometrics of Nellore cattle fed increasing levels of diet starch containing a blend of essential oils and amylase or monensin

**DOI:** 10.3389/fvets.2023.1090097

**Published:** 2023-03-06

**Authors:** Thaiano I. S. Silva, Johnny M. Souza, Tiago S. Acedo, Victor V. Carvalho, Alexandre Perdigão, Leandro A. F. Silva, Antonio M. Silvestre, Maria Betania Niehues, Werner F. Schleifer, Daniel M. Casali, Cyntia L. Martins, Mario D. B. Arrigoni, Danilo D. Millen

**Affiliations:** ^1^Department of Animal Production, College of Agricultural and Technological Sciences, São Paulo State University (UNESP), Dracena, Brazil; ^2^DSM Nutritional Products SA, São Paulo, Brazil; ^3^São Paulo State University (UNESP), School of Veterinary Medicine and Animal Science, Botucatu, São Paulo, Brazil

**Keywords:** additives, concentrate, efficiency, fermentation, non-fiber carbohydrates

## Abstract

Feed additives used in finishing diets improve energy efficiency in ruminal fermentation, resulting in increased animal performance. However, there is no report evaluating the effect of BEO associated with exogenous α-amylase in response to increased starch content in feedlot diets. Our objective was to evaluate increasing levels of starch in the diet associated with a blend of essential oils plus amylase or sodium Monensin on performance, carcass characteristics, and ruminal and cecal morphometry of feedlot cattle. 210 Nellore bulls were used (initial body weight of 375 ± 13.25), where they were blocked and randomly allocated in 30 pens. The experiment was designed in completely randomized blocks in a 3 × 2 factorial arrangement: three starch levels (25, 35, and 45%), and two additives: a blend of essential oils plus α-amylase (BEO, 90 and 560 mg/kg of DM, respectively) or sodium Monensin (MON, 26 mg/kg DM). The animals were fed once a day at 08:00 *ad libitum* and underwent an adaptation period of 14 days. The diets consisted of sugarcane bagasse, ground corn, soybean hulls, cottonseed, soybean meal, mineral-vitamin core, and additives. The animals fed BEO35 had higher dry matter intake (*P* = 0.02) and daily weight gain (*P* = 0.02). The MON treatment improved feed efficiency (*P* = 0.02). The treatments BEO35 and BEO45 increased hot carcass weight (*P* < 0.01). Animals fed BEO presented greater carcass yield (*P* = 0.01), carcass gain (*P* < 0.01), rib eye area gain (*P* = 0.01), and final rib eye area (*P* = 0.02) when compared to MON. The MON25 treatment improved carcass gain efficiency (*P* = 0.01), final marbling (*P* = 0.04), and final subcutaneous fat thickness (*P* < 0.01). The use of MON reduced the fecal starch% (*P* < 0.01). Cattle-fed BEO increased rumen absorptive surface area (*P* = 0.05) and % ASA papilla area (*P* < 0.01). The MON treatment reduced the cecum lesions score (*P* = 0.02). Therefore, the use of BEO with 35 and 45% starch increases carcass production with similar biological efficiency as MON; and animals consuming MON25 improve feed efficiency and reduce lesions in the rumen and cecum.

## 1. Introduction

The increase in performance associated with lowering the age at slaughter is often achieved through the use of high-concentrate feeds to meet energy requirements and maximize production in feedlot cattle (1, 2). The inclusion of grains in diets, as well as the extent of processing, promotes an increase in starch availability and starch breakdown in the rumen, thereby increasing energy intake and rumen fermentation, resulting in increased microbial protein production and high short-chain fatty acid (SCFA) release (3). However, in response to this increased availability of starch in the rumen, negative effects may also occur, such as a decrease in digestibility of fibrous carbohydrates and metabolic disturbances (4). The use of large amounts of starch in conjunction with proper feed management and feed additives is essential for controlling metabolic disorders such as acidosis and bloat (5).

Feed additives used in finishing diets improve energy efficiency in ruminal fermentation, resulting in increased animal performance. Diets with high starch content promote high production of SCFA in the rumen, which leads to a drop in pH. The use of additives is important for controlling rumen acidification and manipulating the rumen fermentation process to maximize the utilization of dietary nutrients (6). Among them, monensin (MON) is widely used in feedlot diets to prevent diseases and metabolic disorders, as well as to improve feed efficiency and animal performance (2, 7). However, the European Union have banned the use of ionophores as they are classified as antibiotics and growth promoters (European Union Regulation, 1831/2003/EC), considering the risk of these products is increasing bacterial resistance to antibiotics and, consequently, possible risks to human health (8).

In this context, this has instigated a search for natural alternatives to ionophores, such as essential oils and their blends (BEO), which act as growth promoters when added to animal feed diets (9). BEO are plant-derived compounds such as thymol, with known antimicrobial, anti-inflammatory, antioxidant, and coccidiostatic properties, that can modulate rumen fermentation to improve nutrient utilization in ruminants (10, 11). The BEO has been reported to make the rumen more energy efficient by increasing the molar ratio of propionate and decreasing the acetate:propionate ratio in the rumen (11), thereby improving nutrient utilization and animal performance (12, 13). BEO can also decrease amino acid deamination and ammonia production in the rumen, improve protein digestibility and promote greater nitrogen retention (11, 14, 15). In this context, some studies have been conducted to evaluate the efficacy of essential oils fed to cattle on animal performance and meat quality (16–18). However, the animal performance was similar in animals that did not receive additives, MON, or BEO. In addition, BEO functionality depends on the source and composition of BEO, as well as the extraction method and dosages used (19, 20). In this context, BEO is an additive with the potential to be utilized in association with monensin or other additives.

It has been suggested that exogenous amylase improves feed efficiency by increasing nutrient utilization, resulting in improved animal performance (18). Some studies reported increased milk production, improved conversion of feed to milk, and increased starch digestion in the rumen (21–23) in response to the administration of exogenous amylase to lactating dairy cows. However, there are few studies investigating the effect of exogenous amylase in finishing cattle (18, 24, 25). Meschiatti et al. (18) reported synergism between BEO and exogenous amylase in a feedlot finishing diet, where the combination of both additives resulted in an increase in DMI, ADG, BW, and hot carcass weight compared to monensin. In addition, BEO in combination with exogenous amylase was reported to reduce liver abscesses and fecal starch in cattle fed high-starch diet (18, 26).

However, based on the aforementioned information, there is no report evaluating the effect of BEO associated with exogenous α-amylase in response to increased starch content in a feedlot diet. It was hypothesized that BEO should be an alternative to MON in finishing feedlot diets, as well as that BEO associated with exogenous enzymes would further improve nutrient utilization and animal performance in high-starch diets, reducing fecal starch and cecum lesions score. Therefore, the objective of this study was to evaluate the increasing starch content in the diet associated with a blend of essential oils plus amylase or sodium monensin on performance, carcass characteristics, and ruminal and cecal morphometry of feedlot cattle.

## 2. Material and methods

All the procedures involving the use of animals in this study were by the guidelines established by the São Paulo State University Ethical Committee for Animal Research, and approved by Ethical Commission in Use of Animals of the Innovation and Applied Science DSM Nutritional Products SA (number BR190313).

### 2.1. Animals and treatments

The trial was conducted at the feedlot facility of the Center for Innovation and Applied Science in Ruminants, from DSM Nutritional Products (I&AS Beef Center; Rio Brilhante, Mato Grosso do Sul, Brazil), using 210 22-mo-old yearlings Nellore bulls (375 ± 13.25 kg). Animals were housed in 30 pens, with 120 m^2^ of area, a water trough, and a collective bunk (50 cm/animal, 5 m of bunk/pen).

The experimental design was performed in completely randomized blocks and the initial BW was utilized as a criterion for block formation, in a 3 × 2 factorial arrangement, in which were evaluated: 3 levels of starch (25, 35, and 45%) and 2 feed additives (BEO + α-amylase or sodium monensin), totaling 6 treatments: (1) MON25: 25% of starch + monensin; (2) BEO25: 25% of starch + essential oil and α-amylase; (3) MON35: 35% of starch + monensin; (4) BEO35: 35% of starch + essential oil and α-amylase; (5) MON45: 45% of starch + monensin; (6) BEO45: 45% of starch + essential oil and α-amylase. Each treatment had 5 replicates (pen as an experimental unit), with 7 animals/pen (total of 30 pens).

The sodium monensin (Rumensin, Elanco, Greenfield, Indiana, USA) was included in the diet at a dose of 26 ppm. The blend of essential oil (Crina ^®^ Ruminants DSM Nutritional Products Ltd., Basel, Switzerland) which contains thymol, eugenol, limonene, and vanillin (14), and the exogenous enzyme α-amylase (Ronozyme Rumistar; DSM Nutritional Products Ltd., Basel, Switzerland) were added to the diet at a dose of 90 and 560 mg/kg of DM, respectively. The doses of feed additives used in this study were to the company's recommendations. It's noteworthy to mention that MON has been largely used around the world, and since results have been very consistent over decades, it was adopted as a positive control in this study.

### 2.2. Feeding and management description

At the beginning of the study, all yearling bulls were weighed, dewormed, and vaccinated (tetanus, bovine viral diarrhea virus, 7-way *Clostridium* sp.; Cattlemaster and Bovishield, Pfizer Animal Health, New York, NY). The animals were submitted to a pre-adaptation period of 10 days to standardize their ruminal population and adapt to the facilities and management. Cattle were fed *ad libitum* once a day at 08:00 h, targeting 3–5% refusal, with free-choice water access to a water trough.

The experimental diets were formulated according to the LRNS (Large Ruminant Nutrition System, Table 1), meeting the nutritional requirements, with daily weight gains between 1.5 and 1.7 kg/day/animal. The experimental diets were composed of sugarcane bagasse, ground corn, soybean hulls, cottonseed, soybean meal, mineral-vitamin core, urea, and additives. The step-up adaptation program diet consisted of *ad libitum* intake and lasted 14 days, whereas 2 adaptation diets containing 65 and 75% concentrate were fed for 7 days each (Table 1). The finishing period program also consisted of *ad libitum* intake and lasted 89 days, where the finishing diet contained 85% concentrate. The different starch content of the diets (25, 35, and 45%) was obtained by increasing the proportion of Corn grain fine grind. During the experimental period, weekly samples were taken of the rations for the chemical analysis of dry matter (DM), crude protein (CP), ether extract (EE), and mineral matter (MM) according to AOAC (27), and neutral detergent fiber (NDF) according to Van Soest et al. (28).

**Table 1:** Feed ingredients and chemical composition of diets containing levels of starch fed to Nellore yearling bulls supplemented with essential oils and amylase or sodium monensin.

**Starch, %^a^**	**25%**			**35%**			**45%**		
**Concentrate, %^b^**	**65%**	**75%**	**85%**	**65%**	**75%**	**85%**	**65%**	**75%**	**85%**
**Ingredients, % of DM^c^**									
Sugarcane bagasse	35	25	15	35	25	15	35	25	15
Corn grain fine grind	30	33	36	30	40	50	30	47	64
Soybean meal	9	5,5	2	9	6,5	4	9	7,5	6
Cottonseed	6	8	10	6	8	10	6	8	10
Soybean hull	15	23,5	32	15	15,5	16	15	7,5	0
Mineral Supplement	5	5	5	5	5	5	5	5	5
**Nutrient content, % of DM^d^**									
Crude protein	14,6	14,7	14,6	14,6	14,7	14,6	14,6	14,5	14,5
Total digestible nutrients	66	68	69	66	69	73	66	72	77
DIP	51	51	50	51	51	52	51	52	53
Neutral detergent fiber	43,7	42,4	41,2	43,7	38,2	33	43,7	31,6	25,2
peNDF^d^	36	30	25	36	29	23	36	28	22
Ca	0,77	0,75	0,73	0,77	0,75	0,73	0,77	0,76	0,75
P	0,31	0,28	0,25	0,31	0,31	0,31	0,31	0,36	0,37
Starch	20,95	23,08	25,46	20,95	28,40	35,50	20,95	37,28	45,80
ME, Mcal/kg DM^e^	2,40	2,44	2,48	2,40	2,51	2,63	2,40	2,62	2,77

a Level of starch (25, 35, and 45%);

b Percent of concentrate of step-up adaptation diets and finishing diet: Adaptation 1 = 65% concentrate, 0–7 days; Adaptation 2 = 75% concentrate, 7–14 days; Finishing diet = 85% concentrate, 14–89 days;

c DM, dry matter;

d Estimated by equations according to Large Ruminant Nutrition System (LRNS; 20).

e Metabolizable energy. DIP, degradable intake protein.

The duration of the experimental period was 99 days, which included a pre-adaptation period of 10 days (day −10 to day 0), a step-up adaptation program diet of 14 days (day 0 to day 14), and a finishing period program of 89 days (day 14 to day 89).

### 2.3. Feedlot performance and carcass traits

At the beginning of the experimental period (day 0), and 30 and 89 days of the study, the feed was withheld from bulls for 16 h before every body weight (BW) assessment. Consequently, average daily gain (ADG), and feed efficiency (gain-to-feed, G/F) were calculated at the end of the experiment. The DMI was calculated for each pen daily, by weighing the ration offered and refused before the next morning delivery and expressed in kilograms and as a percentage of BW. To estimate the net energy for maintenance and net energy for gain, the methods described by Lofgreen and Garrett (29), NRC (30), and Zinn and Shen (31) were used. The values obtained by the equations of net energy for gain were related to the average values estimated by the LRNS, proportional to each diet (25, 35, and 45% starch) and additives evaluated (32).

At the end of the adaptation period, one animal from each block (*n* = 6) was randomly chosen to be slaughtered as a reference to the evaluation of the gain composition in the function of the initial BW, and determination of the equation of adjustment of the BW in the function of the carcass weight (model equation reference: HCW = 12.2581 + 0.4837^*^initial BW). The remaining Nellore yearling bulls were harvested at the end of the study. The final BW was obtained at the feedlot before truck loading, and the cattle were then transported to a commercial abattoir. The hot carcass weight (HCW) was obtained after kidney, pelvic, and heart fat removal. The dressing percentage was calculated by dividing HCW by the final BW (33).

### 2.4. Feeding behavior and particle sorting

All yearling bulls were submitted to visual observations to evaluate feeding behavior, every 5 min, over one period of 24 h (day 71), according to Johnson and Combs (34). Feeding behavior data were recorded for each animal as follows: time spent resting, ruminating, and eating (expressed in minutes), and the number of meals per day. Meal length (expressed in minutes) was calculated by dividing the time spent eating by the number of meals per day. The DMI per meal in kilograms was calculated by dividing DMI by the number of meals per day. In addition, data on time spent eating and ruminating were used to calculate the eating rate of DM (time spent eating / DMI) and rumination rate of DM (time spent ruminating / DMI), both expressed in minutes per kilogram of DM, according to Pereira et al. (35).

During the observation period, samples of diets and orts were collected after 24 h for future analyses of DM [AOAC, (27)] and neutral detergent fiber [NDF; (28)], to determine DMI and NDF intake on the day of the feeding behavior evaluations. Moreover, the eating rate (DM and NDF), as well as the rumination rate (DM and NDF), time spent eating per meal, and dry matter intake per meal was calculated as described by Carvalho et al. (36).

On the day of observation of feeding behavior (day 71), samples of diets and orts were also collected from all pens for the determination of particle size distribution, which was performed by sieving using the Penn State Particle Size Separator (Nasco, Fort Atkinson, WI, EUA) and reported on an as-fed basis as described by Heinrichs and Kononoff (37). Particle sorting was determined as follows: *n* intake/*n* predicted intake, in which *n* = particle fraction retained on screens of 19 (long), 8 (medium), and 1.18 mm (short) and a pan (fine). Particle sorting values equal to 1 indicate no sorting. Those < 1 indicate selective refusal (sorting against), and those >1 indicate preferential consumption sorting for Leonardi and Armentano (38).

### 2.5. Rumen and cecum morphometrics

Rumenitis evaluation was performed after bull evisceration, and all rumens were scored after washing. Rumen epithelium was classified according to the incidence of lesions (rumenitis and hyperkeratosis) as described by Bigham and McManus (39) based on a scale of 0 (no lesions) to 10 (severe ulcerative lesions).

A small fragment (1 cm^2^) of each rumen was collected from the dorsal cranial sac, and placed into a phosphate-buffered saline solution for future morphometric measurements according to Resende Júnior et al. (40). The number of papillae per square centimeter of rumen wall (NOP) was determined manually, where 12 papillae were randomly collected from each fragment (1 cm^2^) and scanned, and the mean papillae area (MPA) was determined using an image analysis system (Image Tool, version 2.01-4; UTHSCSA Dental Diagnostic Science, San Antonio, TX, USA). The rumen wall absorptive surface area (ASA) was calculated according to Daniel et al. (41).

**Figure 1:**
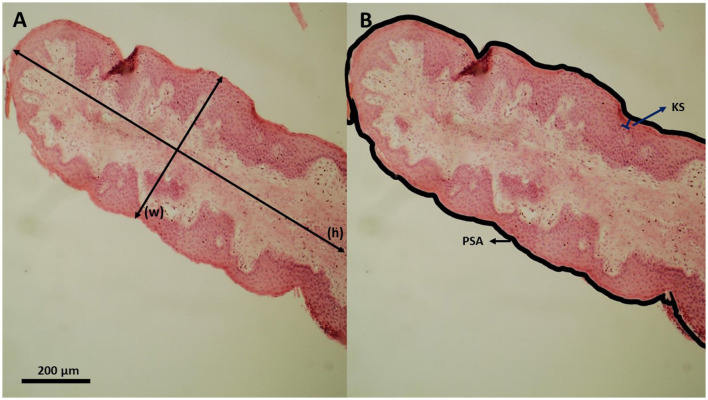
Histological section of a cattle ruminal papilla; **(A)** Papillae height (h) and papillae width (w; measured perpendicular to papilla length). **(B)** Papillae surface area (PSA; thick outer line) and stratum corneum thickness (KS; keratinized surface).

For histological analyzes, a method adapted from Odongo et al. (42) was used. A 1 cm^2^ fragment of each rumen was collected from the ventral cranial sac, stained with hematoxylin and eosin, embedded in paraffin wax, and sectioned. Histological measurements, such as papillae height, papillae width, papillae surface area, and keratinized layer thickness were performed in four papillae per bull using a computer-aided light microscope image analysis. Measurements were performed per animal, in 10% of the total papillae per cm^2^, which were chosen randomly. The final value for each variable was the average of measured papillae (Figure 1).

The same 1-cm^2^ fragment collected from the ventral cranial sac was also used for the evaluation of cell proliferation of rumen papillae according to the immunohistochemistry method adapted from Pereira et al. (43). To determine the mitotic index, the nuclei of 2,000 cells of the basal layer of the ruminal epithelium were marked, and among these, the number of mitotic figures was counted. Thus, the proportion of cells in mitosis in the basal layer of the ruminal epithelium was determined.

Cecum epithelium was classified according to the presence of cecal wall lesions and abnormalities, according to the method adapted from Pereira et al. (44). Histological measurements, such as crypt depth and goblet cells, were determined in 10% of the total number of crypts per animal, using a Leica Qwin Image Analyzer within a Leica electron light microscope.

### 2.6. Shear force analysis and meat color

For meat quality analysis, 2.54-cm thick steak samples of *Longissimus dorsi* (LM) were obtained between the 12th and 13th ribs of animals (3 animals/pen). The samples were identified, vacuum packaged, and frozen at −20°C for further analysis. The analyzes were performed at the Food Products of Animal Origin Laboratory (Economy, Sociology and Technology Department—School of Agriculture UNESP—Botucatu, Brazil).

For the shear force analysis, the samples were thawed, for 24 h at 2°C, and processed according to the American Meat Science Association (AMSA) (45) and Wheeler et al. (46) using a Warner—Bratzler shear force device. The shear force of each sample was considered as the average of 6–8 repetitions (cylinders). The analysis of cooking losses was performed following the methodology described by Honikel (47).

The color was determined by the CIE Lab system (48). Color measurement was performed in three distinct points of the sample, utilizing a spectrophotometer, model CM2500d (Konica Minolta Brazil, São Paulo, Brazil) with D65 illuminating standard, observation angle of 10°, and 30 mm shutter opening. The samples were left exposed to the environment for 20 min for color determination, where L^*^ is the chrome associated with luminosity (L^*^ = 0 black; 100 white), a^*^ is the chrome that varies from green (–) to red (+), and b^*^ is the chrome that ranges from blue (–) to yellow (+). Color values were considered the average of the three readings.

For the evaluation of the proximate composition, samples of *Longissimus dorsi* were used, and thawed the day before the analysis in a refrigerator at 2–5°C. Moisture was evaluated according to AOAC (method 39.1.02) (49). The total nitrogen was quantified using the Kjeldahl method according to AOAC (method 39.1.19) (49). Total protein was calculated as a function of total nitrogen, using the conversion index of 6.25. The ethereal extract was determined according to AOAC (method 39.1.05) (49). Ash was determined according to AOAC (method 39.1.09) (49).

### 2.7. Statistical analysis

The experimental design was performed in completely randomized blocks, and the initial BW was utilized as a criterion for block formation, in a 3 × 2 factorial arrangement of treatments (3 levels of starch and 2 feed additives). Pens were considered the experimental unit for this study (*n* = 30), and each treatment was replicated five times. Data were analyzed using the MIXED procedure of SAS (SAS, 2003) and the Tukey test to compare means. Tests for normality (Shapiro-Wilk and Kolmogorov-Smirnov) and heterogeneity of treatment variances (GROUP option of SAS) were performed before analyzing the data. The model also included fixed effects of treatments and their interaction. The block was considered a random effect in the model. Results were considered significant at *P* ≤ 0.05 level.

## 3. Results

### 3.1. Feedlot performance and carcass traits

The results of feedlot performance are presented in Table 2. A significant interaction between starch level and additives was observed for BW (*P* < 0.01) and ADG (*P* = 0.01) on the first 30 days of the study, where the cattle fed BEO45 had greater values when compared with MON in the same levels of starch. Similarly, there is a significant interaction between starch level and additives for DMI (*P* < 0.01) and DMI expressed as %BW (*P* = 0.01) on the first 30 days of the study; and for final BW (*P* = 0.04), ADG (*P* = 0.02), DMI (*P* = 0.02) and DMI expressed as %BW (*P* = 0.05) at the end of the experimental period, where the cattle fed BEO35 had greater values when compared with MON35 and MON45. No effects of treatments were observed (*P* > 0.05) for G/F in the first 30 days of the study. However, at the end of the study, there was a significant linear decrease in G/F (*P* = 0.05) in response to increasing levels of starch (0.151, 0.149, and 0.145, for 25, 35, and 45% starch, respectively). Moreover, cattle-fed MON improved G:F (0.146 vs. 0.150, for BEO and MON, respectively, *P* = 0.02). Regarding fecal starch, there was a significant (*P* < 0.01) quadratic effect in response to increasing levels of starch, where cattle fed BEO had greater fecal starch content when compared with MON (8.48 vs. 5.90, for BEO and MON, respectively, *P* = 0.05; Table 2).

**Table 2:** Levels of starch associated with a blend of essential oils or sodium monensin on feedlot performance of Nellore yearling bulls.

**Treatments^a^**	**BEO**			**MON**				***P-*** **value^c^**		
**Level of starch, %**	**25%**	**35%**	**45%**	**25%**	**35%**	**45%**	**SEM^b^**	**Additive**	**Starch**	Additive*Starch
Initial BW, kg^d^	375.99	376.02	378.61	374.98	375.14	373.94	5.61	0.17	0.90	0.53
BW 30 days, kg	421.33^b^	428.57^a^	431.86^a^	422.02^a^^b^	418.64^b^	417.39^b^	4.62	0.05	0.47	< 0.01
Final BW, kg	510.35^a^	516.72^a^	510.27^a^	506.45^a^^b^	497.23^b^^c^	487.47^c^	5.68	< 0.01	0.04	0.04
ADG 30 days, kg	1.46^b^	1.70^a^	1.72^a^	1.52^b^	1.40^b^	1.40^b^	0.07	< 0.01	0.49	0.01
ADG, kg/days	1.51^a^^b^	1.58^a^	1.48^b^	1.48^b^	1.37^c^	1.28^d^	0.04	< 0.01	< 0.01	0.02
DMI 30 days, kg/days	9.30^b^	9.92^a^	9.73^a^	9.22^b^	8.92^c^	8.77^c^	0.10	< 0.01	0.16	< 0.01
DMI, kg/days	10.37^a^^b^	10.68^a^	10.29^b^	9.54^c^	9.16^d^	8.76^e^	0.11	< 0.01	< 0.01	0.02
DMI, % of BW 30 days, kg/days	2.33^c^	2.47^a^	2.40^b^	2.31^c^	2.25^d^	2.22^d^	0.12	< 0.01	0.08	0.01
DMI, % of BW	2.34^a^^b^	2.39^a^	2.32^b^	2.16^c^	2.10^c^^d^	2.03^d^	0.02	< 0.01	< 0.01 (L)	0.05
G/F ratio 30 days, kg/kg	0.157	0.171	0.177	0.165	0.158	0.160	0.008	0.19	0.58	0.15
G/F, kg/kg	0.146	0.148	0.144	0.155	0.150	0.146	0.004	0.02	0.05 (L)	0.19
Fecal starch, %	4.94	10.73	9.76	3.47	6.86	7.38	0.64	0.05	< 0.0001 (Q)	0.39

a BEO: blend of essential oil (Crina ^®^ Ruminants and exogenous enzyme α-amylase Ronozyme RumiStar™); MON: monensin;

b SEM = Standard error mean;

c L = linear effect; Q = quadratic effect;

d BW, body weight; G: F, gain-to-feed ratio; ADG, average daily gain; DMI, dry matter intake. Values within a row with different superscripts differ (*P* < 0.05).

The results of carcass traits are presented in Table 3. A significant interaction between starch level and additives was observed for final HCW (*P* = 0.01) and HCW gain (*P* = 0.01), where the cattle fed BEO25 and BEO35 had greater values when compared with MON35 and MON45. However, a significant interaction between starch level and additives was observed for final carcass gain efficiency (*P* = 0,01), biological efficiency (*P* = 0,01), final marbling (*P* = 0,04), final subcutaneous fat thickness (*P* = 0,01) and P8 rump fat thickness gain (*P* = 0,01), where the cattle fed MON25 had greater values when compared to BEO25 and BEO45. Moreover, there was a significant linear decrease in dressing percentage (*P* = 0.01), carcass gains (*P* = 0.05), final P8 rump fat thickness (*P* = 0.01), rib-eye area gain (*P* = 0.03) and P8 rump fat thickness gain (*P* = 0.01) in response to increasing levels of starch. In addition, cattle fed BEO had higher dressing percentage (55.38 vs. 54.56 %, for BEO and MON, respectively, *P* = 0.01), carcass gain (66.78 vs. 64.33 %, for BEO and MON, respectively, *P* = 0.01), rib-eye area (67.72 vs. 65.47 cm^2^, for BEO and MON, respectively, *P* = 0.02) and rib-eye area gain (0.18 vs. 0.16 cm^2^/days for BEO and MON, respectively, *P* = 0.01) when compared with MON.

**Table 3:** Levels of starch associated with *blend of* essential oils or sodium monensin on carcass characteristics of Nellore yearling bulls.

**Treatments^a^**	**BEO**			**MON**				***P-*** **value^c^**		
**Level of starch, %**	**25%**	**35%**	**45%**	**25%**	**35%**	**45%**	**SEM^b^**	**Additive**	**Starch**	Additive*Starch
Initial Hot carcass weight, kg	194.12	194.14	195.39	193.64	193.71	193.13	2.71	0.17	0.90	0.53
Final Hot carcass weight, kg	282.76^a^^b^	287.42^a^	281.38^b^	279.90^b^	270.91^c^	263.00^d^	3.91	< 0.01	< 0.01	0.01
Hot carcass weight gain, kg	1.00^a^^b^	1.06^a^	0.98^b^	0.98^b^	0.88^c^	0.79^d^	0.02	< 0.01	< 0.01	0.01
Carcass gain efficiency, kg/kg	0.097^b^	0.099^a^^b^	0.095^b^^c^	0.102^a^	0.096^b^	0.090^c^	0.001	0.592	0.01	0.01
Dressing, %	55.39	55.62	55.13	55.26	54.48	53.95	0.32	0.01	0.01 (L)	0.07
Carcass gain, %	66.90	67.40	66.03	66.46	64.18	62.34	1.96	0.01	0.05 (L)	0.22
Biological efficiency, kg DM/@	156.8^b^	152.9^b^^c^	160.14^a^^b^	148.18^c^	158.81^b^	167.51^a^	2.05	0.51	0.01	0.01
Initial rib-eye area, cm^2^	50.99	51.16	51.04	50.85	51.16	50.83	0.62	0.88	0.96	0.99
Final rib-eye area, cm^2^	67.91	68.44	66.82	66.52	65.73	64.17	0.73	0.02	0.24	0.79
Initial marbling, %	2.05	2.15	2.22	2.2	1.93	2.26	0.054	0.87	0.07	0.08
Final marbling, %	2.45^b^	2.61^a^^b^	2.64^a^^b^	2.72^a^	2.51^b^	2.65^a^^b^	0.05	0.314	0.47	0.04
Initial sub. fat thickness, mm	2.24	2.25	2.29	2.31	2.27	2.41	0.03	0.03	0.08	0.53
Final sub. fat thickness, mm	3.77^b^	3.95^a^^b^	3.97^a^^b^	4.33^a^	3.81^b^	3.71^b^	0.101	0.64	0.28	0.01
Initial P8 rump fat thickness, mm	4.22	4.26	4.15	4.19	4.21	4.34	0.046	0.44	0.76	0.12
Final P8 rump fat thickness, mm	6.64	6.38	6.37	6.89	6.09	6.08	0.167	0.52	0.01 (L)	0.30
Rib-eye area daily gain, cm^2^/days	0.19	0.19	0.17	0.17	0.16	0.14	0.01	0.01	0.03 (L)	0.49
Sub. fat thickness daily gain, mm/days	0.017^b^^c^	0.019^a^^b^	0.019^a^^b^	0.022^a^	0.017^b^^c^	0.014^c^	0.001	0.86	0.13	0.01
P8 rump fat thickness daily gain, mm/days	0.027	0.023	0.024	0.030	0.021	0.019	0.002	0.36	0.01 (L)	0.14

a BEO: blend of essential oil (Crina ^®^ Ruminants and exogenous enzyme α-amylase Ronozyme RumiStar™); MON: monensin;

b SEM = Standard error mean;

c L = linear effect; Q = quadratic effect; Values within a row with different superscripts differ (*P* < 0.05).

The results of meat color and chemical composition are presented in Table 4. There was a significant quadratic effect for cooking losses (*P* < 0.01) and moisture (*P* = 0.02) in response to increasing levels of starch, where the highest value for cooking loss and the lowest value for moisture was obtained on a 35% starch diet. No effects of treatments were observed (*P* > 0.05) for chroma L, chroma A, chroma B, pH, marbling, shear force, ash, and protein (Table 4).

**Table 4:** Levels of starch associated with blend of essential oils or sodium monensin on *shear force* analysis and meat color of Nellore yearling bulls.

**Treatments^a^**	**BEO**			**MON**				***P-*** **value^c^**		
**Level of starch, %**	**25%**	**35%**	**45%**	**25%**	**35%**	**45%**	**SEM^b^**	**Additive**	**Starch**	Additive*Starch
Chroma L	39.16	39.93	39.06	39.15	39.57	38.95	0.43	0.77	0.55	0.96
Chroma A	18.84	19.34	18.52	18.96	19.69	19.04	0.32	0.42	0.28	0.92
Chroma B	8.57	8.98	8.45	8.58	9.13	8.55	0.20	0.73	0.11	0.98
pH, *n*	5.85	5.71	5.80	5.81	5.77	5.77	0.04	0.85	0.10	0.45
Cooking losses, %	27.93	30.23	26.49	27.75	29.44	28.65	0.55	0.60	< 0.01 (Q)	0.39
Marbling, %	2.08	2.04	1.96	2.18	1.94	1.98	0.09	0.93	0.18	0.55
*Shear force*, kgf	3.98	4.26	4.48	4.31	4.34	4.55	0.14	0.25	0.18	0.62
Moisture, %	75.10	74.59	75.36	74.89	75.01	75.45	0.17	0.56	0.02 (Q)	0.31
Ash, %	1.11	1.08	1.11	1.08	1.14	1.12	0.01	0.47	0.56	0.15
Protein, %	17.65	17.57	17.95	17.85	17.81	17.65	0.12	0.74	0.85	0.30

a BEO: blend of essential oil (Crina ^®^ Ruminants and exogenous enzyme α-amylase Ronozyme RumiStar™); MON: monensin;

b SEM = Standard error mean;

c L = linear effect; Q = quadratic effect; Values within a row with different superscripts differ (*P* < 0.05).

### 3.2. Feeding behavior and particle sorting

The results on feeding behavior are summarized in Table 5. A significant interaction between starch level and additives was observed for feed efficiency (*P* = 0.04), time spent resting (*P* = 0.02), and meal length (*P* =0.02). Regarding feed efficiency, the highest value was observed in cattle fed MON45, followed by MON35 and BEO45. For time spent resting, the highest values were observed for BEO35 and MON45. In addition, for meal length, the lowest values were observed for BEO45 and MON45. Regarding particle sorting, no significant treatment effect was observed (*P* > 0.05) for most of the feeding behavior and particle sorting variables evaluated. However, animals fed CR35 sorted (*P* = 0.02) more intensively for medium diet particles when compared to MON35.

**Table 5:** Levels of starch associated with blend of essential oils or sodium monensin on feeding behavior and particle sorting of Nellore yearling bulls.

**Treatments^a^**	**BEO**			**MON**				***P-*** **value^c^**		
**Level of starch, %**	**25%**	**35%**	**45%**	**25%**	**35%**	**45%**	**SEM^b^**	**Additive**	**Starch**	Additive*Starch
**Feeding behavior**										
DMI, kg^d^	11.34	12.23	11.37	10.67	10.50	9.94	0.23	< 0.01	< 0.01 (Q)	0.09
Feed efficiency of DM,	15.61^b^	15.06^b^	18.43^a^	16.15^b^	19.29^a^	19.60^a^	0.54	< 0.01	< 0.01	0.04
Time spent eating, min	176.43	183.95	208.57	171.71	202.71	194.14	4.80	0.98	< 0.01 (L)	0.07
Time spent ruminating, min	256.00	228.29	250.29	213.86	235.71	210.00	12.13	0.02	0.93	0.11
Time spent resting, min	1,007.57^a^^b^	1,027.76^a^	981.14^b^	1,054.43^a^	1,001.57^a^^b^	1,035.86^a^	14.55	0.05	0.32	0.02
Drinking bouts, *n*	3.24	3.94	3.71	3.57	3.54	3.65	3.61	0.85	0.45	0.43
Meals per day, *n*	15.09	16.80	17.29	13.89	16.31	17.23	0.60	0.42	< 0.01 (L)	0.76
ER of NDF, min/kg DM^e^	37.92	42.95	65.93	38.92	52.55	68.92	2.34	0.09	< 0.01(L)	0.37
RR of DM, min/kg DM	22.75	18.74	21.98	20.19	22.53	21.25	1.16	0.88	0.71	0.06
RR of NDF, min/kg DM^f^	55.13	53.37	78.49	48.69	61.61	74.99	3.92	0.89	< 0.01(L)	0.15
Meal length, min	11.79^a^	11.04^a^^b^	12.25^a^	12.42^a^	12.66^a^	11.31^a^^b^	0.51	0.21	0.72	0.02
DMI per meal, kg	0.76	0.74	0.67	0.78	0.66	0.58	0.03	0.11	< 0.01 (L)	0.35
NDF intake, kg	4.69	4.30	3.21	4.43	3.90	2.87	0.11	0.02	< 0.01(L)	0.91
**Particle sorting^g^**										
Long	1.02	0.92	0.91	1.00	0.87	0.96	0.03	0.92	0.01	0.33
Medium	1.01^a^	0.99^a^	0.94^b^	1.00^a^	0.94^b^	0.97^a^^b^	0.01	0.40	< 0.01	0.02
Short	1.00	1.01	1.02	1.00	1.01	1.01	0.00	0.40	0.01	0.06
Fine	0.99	1.01	1.01	1.00	1.02	1.01	0.0045	0.26	0.03	0.40

a BEO: blend of essential oil (Crina ^®^ Ruminants and exogenous enzyme α-amylase Ronozyme RumiStar™); MON: monensin;

b SEM = Standard error mean;

c L = linear effect; Q = quadratic effect;

d DMI, dry matter intake;

e ER, eating rate;

f RR, rumination rate;

g Particle fraction retained on screens of 19 (long), 8 (medium), 1.18 mm (short) and a pan (fine). Values within a row with different superscripts differ (*P* < 0.05).

There was a significant quadratic effect for DMI (*P* < 0.01) in response to increasing levels of starch, where the highest value was obtained on 35% starch (11.01, 11.37, and 10.66 kg/days for 25, 35, and 45% starch, respectively). Moreover, there was a significant linear increase in time spent eating (*P* < 0.01), meals per day (*P* < 0.01), eating rate of NDF (*P* < 0.01), rumination rate of NDF (*P* < 0.01), in response to increasing levels of starch. There was a significant linear decrease in DMI per meal (*P* < 0.01) and eating rate of NDF (*P* < 0.01) in response to increasing levels of starch.

Besides that, DMI (11.65 vs. 10.37 kg/days, for BEO and MON, respectively; *P* < 0.01), time spent ruminating (244.86 vs. 219.86 min, for BEO and MON, respectively; *P* = 0.02) and NDF intake (4.07 vs. 3.73 kg, for BEO and MON, respectively; *P* = 0.02) were affected by the type of additive, where cattle fed BEO obtained the best results. No effects of treatments were observed (*P* > 0.05) for the rumination rate of DM and drinking bouts.

### 3.3. Rumen and cecum morphometrics

The results on rumen and cecum morphometrics are presented in Table 6. There was a significant quadratic effect for rumenitis score (*P* = 0.03) in response to increasing levels of starch, where the highest value was obtained on 35% starch (0.80, 1.05, and 0.88 for 25, 35, and 45% starch, respectively).

**Table 6:** Levels of starch associated with blend of essential oils or sodium monensin on rumen and cecum morphometrics of Nellore yearling bulls.

**Treatments^a^**	**BEO**			**MON**				***P-*** **value^c^**		
**Level of starch, %**	**25%**	**35%**	**45%**	**25%**	**35%**	**45%**	**SEM^b^**	**Additive**	**Starch**	Additive*starch
Rumenitis score	0.85	1.00	0.95	0.75	1.10	0.80	0.06	0.50	0.03 (Q)	0.36
**Macroscopic variables**										
Mean papillae area, cm^2^	0.52	0.50	0.46	0.48	0.45	0.47	0.02	0.26	0.40	0.40
Number of papillae, *n*	63.98	69.77	70.13	62.33	64.58	64.51	2.63	0.18	0.45	0.84
ASA, cm^2^/cm^2^ of rumen wall^d^	32.82	35.24	30.29	30.23	29.50	30.13	1.13	0.05	0.46	0.28
Papillae area, % of ASA	97.09	97.07	97.02	96.82	96.64	96.77	0.09	0.01	0.76	0.78
**Microscopic variables**										
Mitotic index, *n*	159.73	170	155.4	183.93	162.87	165.93	8.50	0.39	0.69	0.49
Mitotic index, %	7.99	8.50	7.77	9.20	8.14	8.30	0.42	0.39	0.69	0.49
Papillae height, mm	4.18	4.17	4.24	4.04	4.04	3.83	0.08	0.03	0.78	0.41
Papillae surface area, mm3	1.02	0.96	1.01	1.01	0.94	0.92	0.02	0.14	0.22	0.29
Papillae width, mm	0.18	0.16	0.15	0.16	0.16	0.17	0.01	0.78	0.55	0.19
Keratinized layer thickness, μm	7.74	8.29	9.26	7.92	8.04	7.86	0.22	0.09	0.12	0.07
**Cecum variables**										
Cecum lesions score, *n*	1.61	2.55	2.16	1.30	1.43	1.55	0.21	0.02	0.25	0.47
Crypt depth, μm	75.48	76.57	74.88	74.40	76.06	74.17	1.41	0.65	0.73	0.99
Enterocytes, *n*	16.25	16.05	15.95	16.93	16.57	16.51	0.35	0.18	0.77	0.99
Goblet cells, *n*	1.51	1.48	1.45	1.67	1.65	1.65	0.06	0.06	0.94	0.98
Crypt/Enterocytes	4.66	4.79	4.70	4.43	4.60	4.51	0.06	0.01	0.27	0.97
Crypt/Goblet, *n*	50.99	52.21	52.05	46.77	48.56	47.19	1.28	0.02	0.76	0.94

a BEO: blend of essential oil (Crina ^®^ Ruminants and exogenous enzyme α-amylase Ronozyme RumiStar™); MON: monensin;

b SEM = Standard error mean;

c L = linear effect; Q = quadratic effect;

d ASA: absorptive surface area. Values within a row with different superscripts differ (*P* < 0.05).

Moreover, animals fed BEO obtained greater absorptive surface area (ASA; 32.78 vs. 29.95 cm^2^/cm^2^ of rumen wall, for BEO and MON, respectively; *P* = 0.05), papillae area (97.06 vs. 96.74% of ASA, for BEO and MON, respectively; *P* = 0.01) and papillae height (4.20 vs. 3.97, for BEO and MON, respectively; *P* = 0.03) when compared to MON treatment. However, the supplementation with BEO increased the cecum lesions score (2.11 vs. 1.43, for BEO and MON, respectively; *P* = 0.02), crypt/enterocytes ratio (4.72 vs. 4.51, for BEO and MON, respectively; *P* = 0.01) and crypt/goblet ratio (51.75 vs. 47.51, for BEO and MON, respectively; *P* = 0.02).

## 4. Discussion

Essential oils (EO) and exogenous enzymes may have the potential to replace ionophores, increase BW and ADG and contribute to improved beef production and animal performance (50), but the amount of research information on the potential benefits of these new technologies compared to the most common feed additives for feedlot cattle is limited (18). In this context, the present study answers new questions regarding the use of BEO, evaluating the effect of essential oil associated with exogenous α-amylase in response to increased starch content in a feedlot diet. The EO are aromatic oily liquids extracted from plant material that possess a broad spectrum of antimicrobial activities (11, 51). However, a detailed explanation of how EO affects ruminal fermentation by altering microorganisms has not been established (18, 50).

In the present study, cattle fed BEO35 had the highest final BW and ADG, and DMI compared with treatments at MON; however, the results for feed efficiency were more satisfactory compared with treatments fed BEO regardless of starch content than when MON was used. These results could be related to the change in rumen fermentation pattern in response to essential oil treatment in combination with exogenous α-amylase, which may alter the acetate/propionate ratio in the rumen. However, results regarding DMI in response to supplementation with EO are inconsistent because several factors may influence the mode of action of this additive, such as the type of feed, breed, sex, and age, as well as the type of essential oil used in the blend (18, 52). Essential oils are plant-derived compounds with known antimicrobial, anti-inflammatory, antioxidant, and coccidiostatic properties. In addition, EO has properties similar to ionophores and contains compounds that have been shown to modulate rumen fermentation by selecting microorganisms in the rumen, thereby improving nutrient utilization in ruminants (53). The BEO might have a flavor effect on feed which may have contributed to increase DMI, as was previously reported (54). In addition, the association of EO with α-amylase, such as BEO, ensures the better breakdown of glycosidic bonds present in starch, which serve as a substrate and consequently increase the abundance of non-fibrous carbohydrate-fermenting bacteria. Salazar et al. (54) reported greater DMI for cattle receiving BEO when compared to MON. Moreover, Tricarico et al. (55) reported an increase in DMI when exogenous α-amylase was used due to a change in the molar ratio of butyrate and propionate, and a decrease in rumen lactate concentration. In this context, previously study reported the negative effects of increasing ruminal propionate on DMI in dairy cows (56). This may justify the increase in DMI in BEO-treated animals, which may have increased the rumen passage rate.

In this context, data on feeding behavior may help in the interpretation of DMI in response to BEO supplementation. However, evaluation of the feeding behavior in feedlot cattle supplemented with BEO is limited in the literature (57, 58). In the present study, the variables examined were significantly affected by starch content. Cattle fed BEO35 sorted for medium diet particles and spent more time feeding NDF and ruminating NDF, which may be a response to control rumen acidity and a possible explanation for the increased DMI of cattle consuming BEO. In addition, increasing the starch content of the diet resulted in more meals per day and lower DM intake per meal while attempting to control rumen acidosis. Moreover, essential oils may decrease protein breakdown in the rumen, which promotes less colonization by bacteria with proteolytic activity (59). This increase in rumination rate in response to BEO treatment may have a positive effect on reducing the size of feed particles and increasing the contact area for bacteria and rumen fermentation. On the other hand, longer rumination times may affect feed passage rate and, thus, DMI.

Regarding monensin, it is well documented that the main effects of ionophores are a reduction in DMI and increased feed efficiency (6, 7, 33). A meta-analysis by Duffield et al. (7) reported that the use of monensin decreased DMI by 3% and improved feed efficiency by 2.5–3.5% in feedlot beef cattle. This higher DMI of BEO-supplemented animals may also be attributed to the essential oils' palatability. Depending on the plants extracted, some essential oils have active ingredients that can improve the palatability of the diet (60). Meschiatti et al. (18) reported higher DMI in Nellore feedlot cattle fed a 55% starch diet associated with the use of a BEO plus exogenous α-amylase compared with a treatment containing only essential oils or monensin. However, Meyer et al. (50) found no difference in DMI in feedlot cattle receiving a blend of essential oils.

In this context, starch-rich diets can increase the risk of rumen acidosis, as well as lesions in the ruminal epithelium, due to the greater rumen fermentation and consequently higher production of SCFA (33). The number of papillae, papillae area, and absorptive surface area (ASA) are important parameters directly related to the absorption of SCFA, preventing the accumulation of these acids in the rumen environment and acidification (17, 61). In the present study, the highest rumenitis score was observed in diets containing 35% starch, regardless of the additive. On the other hand, supplementation with BEO increased the papillae height and papillae area (% of ASA). Due to the higher DMI observed in response to BEO, this treatment increased the production of SCFA and, consequently, resulted in greater papillae development. In addition, the use of essential oil may act as a modulator of rumen fermentation by selecting lactic acid-producing bacteria and protecting the rumen from a possible drop in rumen pH, due to the high amounts of fermented starch (62). Li et al. (63) reported that the use of a BEO altered rumen fermentation with increased propionate concentration, improved fiber digestibility, and decreased methane production.

However, treatment with BEO also increased the cecum lesions score and decreased the number of goblet cells. Considering the higher DMI observed, it may be that animals fed BEO had a higher rumen passage rate so that a greater amount of starch bypassed the rumen and some of this starch entered the cecum, which may lead to excessive fermentation in the cecum, increasing the production and accumulation of SCFA, and lowering the pH in the cecum. Thus, acidification of the intestinal environment may lead to an increase in lesions in the cecal epithelium and a decrease in the number of goblet cells and enterocytes because the intestinal epithelium is more sensitive to pH changes than the rumen (64). However, in a companion study, Rocha et al. (65) reported that cattle fed BEO plus amylase surprisingly did not have proteins related to inflammatory processes (leukocyte elastase inhibitors) in the cecal tissues, although cecal lesions increased in response to BEO. This could be because feed enzymes remain active in the intestine and can help digest nutrients that escape rumen fermentation, or they may have even had an anti-inflammatory effect, but they cannot control cecal lesions.

Just as SCFA concentrations and rumen pH cause changes in SCFA absorption rate (17, 66), SCFA act as signaling and regulatory molecules for biological processes to protect gut health (67). Alteration of goblet cell differentiation, such as expression of mucin-related genes, is associated with increased acetate and propionate concentrations (68). The intestinal epithelium was mechanically stimulated to secrete mucus, which they attributed to the effect of SCFAs on mucus thickening (69). The use of high-starch diets for cattle can lead to lesions and an inflammatory response in the cecum (65). Studies evaluating cecal morphometric parameters, cecal epithelial health, and the correlation of these parameters with animal performance are limited in the literature (44, 70). It has been previously reported in the literature that diet and infusion of fatty acids affect mitotic index, crypt depth, and intestinal mucosal weight in rats (71–74). In the present study, the higher DMI in animals supplemented with BEO may have contributed to this increase in cecum acidity in response to a greater amount of starch fermentation. On the other hand, the higher DMI could have caused a higher production of SCFA, which would develop the cecum and make it less susceptible to inflammatory processes (33, 44). The increase in dietary starch content may promote the down-regulation of enzymes related to carbohydrate degradation, likely caused by damage to the cecal epithelium due to increased responses associated with inflammatory injury (65).

In addition, an increase in fecal starch was observed in animals fed high starch levels, with cattle fed BEO35 having higher fecal starch, suggesting that the cecum was unable to digest the starch from the rumen and absorb SCFA, thereby increasing fecal starch. On the other hand, animals receiving MON had the lowest levels of fecal starch, which may be related to the effectiveness of this additive in influencing rumen fermentation, selection, and stimulating the growth of rumen bacteria, thereby preventing starch losses *via* feces. Ionophore antibiotics have limited effects on the microbiota and intestinal fermentation of ruminants, as the effect of monensin is more likely to be observed in the rumen (65). There are reports that BEO can affect the colonization of starch-rich substrates by rumen bacteria, thereby increasing the availability and utilization of starch in the rumen (75). In the present study, it was observed that BEO did not improve the utilization of dietary starch. Although, as diet starch levels increased, an increase in performance would be expected, as a result of greater diet energy content (Table 1). However, we had no increase in performance as the starch levels increased for BEO, and for MON we had a decrease in performance and intake as starch levels increased. This indicates that we had a non-optimal condition for digestion as starch levels increased, which may have affected animals' digestibility. Meschiatti et al. (18) reported that fecal starch concentration was 25.6% lower and total tract starch digestibility was 5.11% higher (*P* = 0.04) with BEO than with MON.

Thus, the manipulation of rumen fermentation and better feed efficiency, in response to additives use may affect carcass characteristics. In the present study, it was observed that cattle fed BEO35 had the best HCW values. However, the best results in final fat thickness were obtained when cattle were treated with MON25. As mentioned earlier, it was observed that increasing the starch content in the diet resulted in more meals per day and lower DMI per meal while trying to control rumen acidity. As a result, carcass fat deposition was negatively affected in cattle-fed MON because increasing starch content linearly decreased final fat thickness and daily fat gain. Better dressing percentage and carcass gain were observed in cattle fed BEO, which may be explained by the higher DMI observed in this treatment as a function of manipulation of rumen fermentation, and the higher carcass yields may be related to higher production of propionate in the rumen, which is an SCFA precursor of glucose in ruminants (76). Tricarico et al. (55) reported an increase in HCW in feedlot cattle-fed diets supplemented with α-amylase and attributed these results to the improvement in the molar ratio of acetate and propionate and the decrease in lactate. Several active compounds present in essential oils such as carvacrol, thymol, allicin, and pinene have an antimicrobial activity that can affect gram-positive and gram-negative bacteria (77). Because of their ability to modulate the rumen microbiota, essential oils can directly affect protein degradation and SCFA production (78). Meschiatti et al. (18) reported higher HCW in animals fed BEO on a finishing diet containing 55% starch when compared to MON, but no significant difference in dressing percentage was observed. In the present study, the feedlot cattle fed BEO had better results for the rib-eye area, as well as a greater rib-eye area gain, which can be explained by the higher values of HCW and dressing found in these treatments, where greater carcass deposition is a consequence of a greater muscle tissue production.

The change in rumen fermentation pattern in response to the use of these additives affects the production of SCFA and, consequently, the acetate:propionate ratio. There are reports that glucose is the major contributor to fatty acid biosynthesis in intramuscular adipose tissue (79, 80). Considering that propionate is the main substrate for glucose formation in ruminants, maximizing its production in the rumen environment seems to be essential to improve marbling (76). In this context, using feed additives and increasing the availability of starch for rumen fermentation can increase propionate production in the rumen and positively influence intramuscular fat deposition. In the present study, the effect of feed additives on final marbling and final fat thickness was dependent on starch content, where the MON25 presented the best results. In this context, different starch levels associated with this additive may increase fat deposition, leading to better carcass quality. However, Ornaghi et al. (58) reported no differences in final fat thickness, rib-eye area and final marbling in feedlot cattle fed a diet containing 41% starch supplemented with essential oil. Regarding meat quality, higher cooking loss, and lower moisture were observed for treatments BEO35 and MON35. The other quality variables were not affected by the treatments, and the values found in this study are within what is considered normal in the literature (26). Cooking losses are related to intramuscular fat, which has a positive effect on water retention capacity and prevents fluid loss after cooking (81). Meat maturation time is also related to low water loss, which is due to a slight increase in pH and the change from divalent to monovalent ions (82). In general, the average values found for cooking loss vary between 20 and 28% (8, 83), similar to the values obtained in the present study. Toseti et al. (26) observed no difference in cooking loss, pH, shear force, and chroma A and B in response to treatment with BEO.

## 5. Conclusion

The BEO increased DMI and enhanced overall animal performance but the effect was dependent on starch content, with no effect on feed efficiency. Animals receiving monensin improved feed efficiency and decreased lesions in the rumen and cecum in finishing feedlot cattle. Therefore, the blend of essential oil evaluated in the current study can be an alternative to replacing MON in finishing feedlot diets. Overall, cattle fed MON25 improved feed efficiency, whereas BEO presented better results on feedlot performance and carcass traits with 35 and 45% starch diets.

## Data availability statement

The raw data supporting the conclusions of this article will be made available by the authors, without undue reservation.

## Ethics statement

The animal study was reviewed and approved by São Paulo State University Ethical Committee for Animal Research, and approved by Ethical Commission in Use of Animals of the Innovation and Applied Science DSM Nutritional Products SA (number BR190313).

## Author contributions

TS and DM: conceived and designed the study, collected and complied, and analyzed data. TS, TA, VC, AP, LS, AS, MN, WS, and DC: investigation. CM and MA: provided intellectual input. TS, JS, and DM: provided intellectual input, drafted, and edited manuscript. All authors contributed to the article and approved the submitted version.
